# Is the sFlt-1/PlGF ratio efficient in predicting adverse neonatal outcomes in small-for-gestational-age newborns? A prospective observational multicenter cohort study

**DOI:** 10.3389/fmed.2024.1414381

**Published:** 2024-06-10

**Authors:** Katarzyna Kosińska-Kaczyńska, Katarzyna Chaberek, Natalia Szymecka-Samaha, Robert Brawura-Biskupski-Samaha, Agnieszka Czapska, Kinga Żebrowska, Norbert Dera, Jan Madzelewski, Jakub Góra, Kacper Borawski, Weronika Włoch, Anna Scholz

**Affiliations:** ^1^Department of Obstetrics, Perinatology and Neonatology, Center of Postgraduate Medical Education, Warsaw, Poland; ^2^1st Department of Obstetrics and Gynecology, Center of Postgraduate Medical Education, Warsaw, Poland; ^3^Students’ Association, Medical University of Warsaw, Warsaw, Poland

**Keywords:** small-for-gestational-age, fetal growth restriction, neonatal outcome, placental growth factor, soluble fms-like tyrosine kinase-1

## Abstract

**Introduction:**

Fetuses with growth abnormalities are at an increased risk of adverse neonatal outcomes. The aim of this study was to investigate if placental growth factor (PlGF), soluble fms-like tyrosine kinase-1 (sFlt-1), or the sFlt-1/PlGF ratio were efficient predictive factors of adverse neonatal outcomes in small-for-gestational-age (SGA) newborns.

**Methods:**

A prospective observational multicenter cohort study was performed between 2020 and 2023. At the time of the SGA fetus diagnosis, serum angiogenic biomarker measurements were performed. The primary outcome was an adverse neonatal outcome, diagnosed in the case of any of the following: <34 weeks of gestation: mechanical ventilation, sepsis, necrotizing enterocolitis, intraventricular hemorrhage grade III or IV, and neonatal death before discharge; ≥34 weeks of gestation: Neonatal Intensive Care Unit hospitalization, mechanical ventilation, continuous positive airway pressure, sepsis, necrotizing enterocolitis, intraventricular hemorrhage grade III or IV, and neonatal death before discharge.

**Results:**

In total, 192 women who delivered SGA newborns were included in the study. The serum concentrations of PlGF were lower, leading to a higher sFlt-1/PlGF ratio in the adverse outcome group. No significant differences in sFlt-1 levels were observed between the groups. Both PlGF and sFlt-1 had a moderate correlation with adverse neonatal outcomes (PlGF: R − 0.5, *p* < 0.001; sFlt-1: 0.5, *p* < 0.001). The sFlt-1/PlGF ratio showed a correlation of 0.6 (*p* < 0.001) with adverse outcomes. The uterine artery pulsatility index (PI) and the sFlt-1/PlGF ratio were identified as the only independent risk factors for adverse outcomes. An sFlt-1/PlGF ratio of 19.1 exhibited high sensitivity (85.1%) but low specificity (35.9%) in predicting adverse outcomes and had the strongest correlation with them. This ratio allowed the risk of adverse outcomes to be assessed as low with approximately 80% certainty.

**Discussion:**

The sFlt-1/PlGF ratio seems to be an efficient predictive tool in adverse outcome risk assessment. More studies on large cohorts of SGA-complicated pregnancies with and without preeclampsia are needed to develop an optimal and detailed formula for the risk assessment of adverse outcomes in SGA newborns.

## Introduction

The evaluation of fetal growth is one of the essential aspects of perinatal care. The prediction of adverse neonatal outcomes may be based on fetal growth. A fetus small-for-gestational-age (SGA) is usually defined as one with an estimated weight below the 10th centile for gestational age (1). Abdominal circumference below the 10th centile of the given reference ranges is another diagnostic criterion (1). Both definitions are widely accepted and used. Conversely, fetal growth restriction (FGR) is a condition when the fetus is unable to reach its genetic growth potential. It may be related to a variety of causes, including genetic conditions, maternal disease, nutrition, smoking or drug use, intrauterine infections, or placental insufficiency. Due to the complexity of this condition, its definition and diagnosis are much more difficult. An expert consensus on a definition for early and late FGR according to the Delphi procedure was determined in 2016. Based on the consensus, early FGR (below 32 weeks of gestation) is diagnosed in cases of the occurrence of three solitary parameters: abdominal circumference (AC) <3rd centile, estimated fetal weight (EFW) <3rd centile, and absent end-diastolic flow in the umbilical artery (UA); or four contributory parameters (AC) or EFW <10th centile combined with the pulsatility index (PI) >95th centile either in the UA or the uterine artery (UtA). For late FGR (≥32 weeks of gestation), two solitary parameters (AC or EFW <3rd centile) and four contributory parameters (EFW or AC <10th centile, AC or EFW crossing centiles by at least two quartiles on growth charts, and the cerebroplacental ratio < 5th centile or UA-PI >95th centile) were defined (2). Although the definition is detailed and unambiguous, it is based on ultrasound measurements and therefore may be biased (for example, due to fetal weight calculations related to different formulas and human errors). If the risk assessment was strictly based on the differentiation between SGA and FGR, it could be understated for fetuses with an estimated weight at the 5th centile or overestimated for fetuses with a weight at the 3rd centile.

Fetuses with growth abnormalities are at increased risk of adverse neonatal outcomes. Regarding adverse outcomes (AOs), the differences between SGA and FGR are not so obvious. Growth-restricted newborns suffer from numerous pathologies, both in the neonatal period and later in life. Early consequences of growth restriction include cardiovascular conditions (hypotension, persistent fetal circulation, structural heart changes, vessel wall rigidity, cardiac function issues, late systemic hypertension, or secondary pulmonary hypertension), respiratory conditions (an increased need for respiratory support, meconium aspiration syndrome, pulmonary hemorrhage, and bronchopulmonary dysplasia), neurological issues (perinatal asphyxia, microcephaly, cranial ultrasound abnormalities, white matter and gray matter changes on MRI, general movement assessment abnormalities, and EEG abnormalities), and many others (e.g., hypoglycemia, hypocalcemia, hypothermia, sepsis, jaundice, polycythemia, feeding intolerance, necrotizing enterocolitis, renal tubular injury, and retinopathy of prematurity) (3). Regrettably, SGA newborns experience AOs as well. SGA preterm newborns are at a higher risk of intraventricular hemorrhage (OR 3.23, 95% CI 1.2–8.68) or patent ductus arteriosus (OR 2.5, 95% CI 1–6.15) in comparison with appropriate for gestational age preterm infants (4). In low-risk women delivering at term, SGA infants are at a higher risk of severe acidosis at birth, 5 min Apgar score < =3, and Neonatal Intensive Care Unit (NICU) admission (5). SGA newborns delivered beyond 39 weeks are at a higher risk of perinatal death (OR 3.39, 95% CI 1.58–7.27), neonatal death (OR 7.69, 95% CI 1.93–30.58), or serious composite neonatal morbidity (OR 1.21, 95% CI 1.06–1.38) (5). Long-term health consequences are also observed. Fetal cardiovascular programming occurs in SGA fetuses regardless of the Doppler finding, leading to a higher risk of cardiovascular disorders (6). According to Alda et al., being SGA at preterm birth identified adverse neonatal outcomes more reliably than the antenatal suspicion of FGR (7).

In the case of perinatal counseling, neonatal outcome is much more important than a strict definition of fetal growth abnormality or a distinction between SGA age and FGR. Therefore, other factors are being examined for their usefulness in the prediction of AO in SGA newborns. The aim of this study was to investigate if placental growth factor (PlGF), soluble fms-like tyrosine kinase-1 (sFlt-1), and the sFlt-1/PlGF ratio were efficient predictive factors of adverse neonatal outcomes in SGA newborns without differentiating between SGA and FGR.

## Materials and methods

A prospective observational cohort study was performed at the Department of Obstetrics, Perinatology, and Neonatology and the First Department of Obstetrics and Gynecology of the Center of Postgraduate Medical Education in Warsaw between 2020 and 2023. Women who were referred to the departments due to the suspicion of SGA fetuses were counseled. In addition to routine clinical care performed according to the local guidelines, they had blood samples collected for sFlt-1, PlGF, and sFlt-1/PlGF ratio at the time of admission. sFlt-1 and PlGF values were determined using the electrochemiluminescence method (ECLIA) on the Cobas e6000 analyzer using the Elecsys^®^ reagent kits. Moreover, every woman underwent serial ultrasound scans performed every 1 or 2 weeks, cardiotocography above 26 weeks of gestation, and a routine biochemistry panel: a complete blood count, aspartate aminotransferase, alanine aminotransferase, creatinine and uric acid, coagulation system tests, and proteinuria assessment. The ultrasound scan included the estimation of fetal weights, UA, middle cerebral artery (MCA), ductus venosus (DV), and UtA Doppler assessment. The estimated fetal weight (EFW) was calculated using the Hadlock formula (8).

The immediate delivery criteria were repeated persistent, unprovoked fetal heart rate decelerations on cardiotocograph (CTG) or

- at 26 + 0 to 28 + 6 weeks of gestation: DV a-wave at or below baseline or fetal heart rate short-term variation (STV) <2.6 ms;
- at 29 + 0 to 31 + 6 weeks: DV a-wave at or below baseline or STV <3.0 ms;
- at 32 + 0 to 33 + 6 weeks: UA reversed or absent end-diastolic flow or STV <3.5 ms;
- beyond 34 + 0 weeks: UA reversed or absent end-diastolic flow or STV <4.5 ms.

In other cases, delivery was planned at 38 weeks in cases of EFW <10th centile in the absence of any other abnormalities or disorders. Antenatal corticosteroids were administered (intramuscular betamethasone 12 mg 24 h apart) if delivery was anticipated between 24 + 0 and 33 + 6 weeks of gestation. Maternal magnesium sulfate prophylaxis (an intravenous infusion of a 4-g bolus over 20 min followed by 1 g per hour for up to 24 h) was given for fetal neuroprotection if birth before 32 weeks was likely to occur.

The study included women who delivered singleton SGA newborns at any of the two departments and gave written informed consent to participate. The birth weight centile was estimated according to the Fetal Medicine Foundation fetal and neonatal population weight charts (9). The inclusion criteria were as follows: age over 18 years, singleton pregnancy beyond 24 weeks of gestation, birth weight below the 10th centile for gestational age, verified gestational age, live birth, available laboratory test results, and complete medical data on the pregnancy outcome. Gestational age was calculated based on the first day of the last menstrual period or the day of transfer for assisted reproductive technique procedures and verified by crown-rump length measured at the first-trimester ultrasound. Cases with incomplete medical data, no serum angiogenic biomarker test results available, no ultrasound scan performed between 11 and 14 weeks of gestation, and with genetic or major anatomical abnormalities in the fetus were excluded. Gestational hypertension and preeclampsia were diagnosed according to the guidelines of the Polish Society of Obstetricians and Gynecologists (10). Newborns were followed up until discharge. The analysis included birth weight, Apgar score at 5 min after birth, umbilical artery blood pH, hospitalization at the NICU, respiratory disorders requiring continuous positive airway pressure (CPAP) or mechanical ventilation, sepsis, necrotizing enterocolitis (NEC), and intraventricular hemorrhage (IVH) rates.

Adverse neonatal outcome was the primary outcome. It was diagnosed in the case of any of the following:

- <34 weeks of gestation: mechanical ventilation, sepsis, NEC, IVH grade III or IV, and neonatal death before discharge home,
- ≥34 weeks of gestation: NICU hospitalization, mechanical ventilation, CPAP, sepsis, NEC, IVH grade III or IV, and neonatal death before discharge home.

The study protocol was approved by the ethics committee at the Center of Postgraduate Medical Education (no. 36/PB/2020) and was conducted according to the Declaration of Helsinki.

The Mann–Whitney test and Fisher’s exact test were used for the statistical analysis. *p*-values <0.05 were considered significant. Cutoff points were estimated based on ROC curves. Sensitivity, specificity, positive and negative predictive values, and positive and negative likelihood ratios were calculated for the analyzed tests. Multivariate logistic regression analysis was performed to adjust for confounding factors, and adjusted odds ratios (aORs) were calculated. The data were analyzed using Statistica version 13.1.

## Results

**Figure 1:**
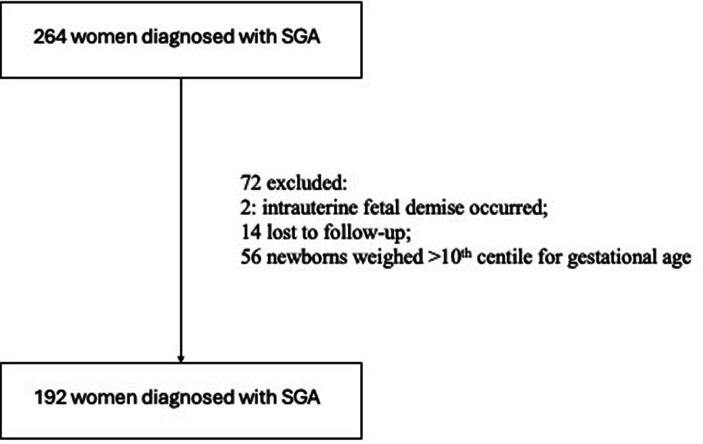
Flowchart of the study group.

During the study period, 264 women were included in the diagnosis of an SGA fetus on ultrasound. A total of 72 were excluded due to several reasons: intrauterine fetal demise occurred in 2 cases, 14 were lost to follow-up, and 56 newborns weighed >10th centile for gestational age. Finally, a total of 192 women who delivered SGA newborns were included in the study. In total, 88 were delivered at the Department of Obstetrics, Perinatology, and Neonatology, and 104 were delivered at the First Department of Obstetrics and Gynecology of the Center of Postgraduate Medical Education. The flowchart is presented in Figure 1. The basic characteristics of the study group are presented in Table 1. All the women were Caucasian. Hypertension was diagnosed in 53.6% of the subjects, which, according to the guidelines of the Polish Society of Obstetricians and Gynecologists, implies a diagnosis of preeclampsia (10).

**Table 1:** Characteristics of the study group.

Characteristics	Study group *n* = 192 median (IQR)	Adverse outcome group *n* = 75 median (IQR)	No adverse outcome group *n* = 117 median (IQR)	*p*
Age (years)	32 (29–35)	31 (28–36)	32 (29–35)	0.8
Primiparous*	122 (63.5)	43 (57.3)	79 (67.5)	0.3
Smoking*	26 (13.5)	12 (16)	18 (15.4)	0.9
Hypertension*	103 (53.6)	45 (69)	58 (49.6)	0.2
ALT (IU/L)	17.1 (11.5–34.9)	16.5 (10.5–41.2)	17.6 (12.5–31.5)	0.8
AST (IU/L)	21.1 (15.5–30.8)	23.2 (16.6–34)	20.6 (15–29.1)	0.2
Creatinine (mg/dL)	0.77 (0.6–0.9)	0.8 (0.7–0.9)	0.71 (0.6–0.9)	0.2
LDH (U/L)	297.7 (170–406.7)	324.1 (189–421)	275.4 (161–403.8)	0.3
Proteinuria*	25 (13)	9 (12)	16 (13.7)	0.9
PLT (G/L)	187 (152–220.7)	190 (150–225)	187 (153–219)	0.8
UtA PI*	1.17 (0.8–1.7)	1.36 (0.9–1.9)	1.04 (0.7–1.6)	0.04
UA PI*	1.29 (1–1.6)	1.4 (1.2–1.6)	1.18 (0.9–1.6)	0.01
AEDF*	20 (10.4)	5 (6.7)	15 (12.8)	0.2
REDF*	9 (4.7)	4 (5.3)	5 (4.3)	0.8
MCA PI*	1.46 (1.3–1.7)	1.43 (1.2–1.8)	1.48 (1.3–1.7)	0.6
DV PIV*	0.55 (0.4–0.8)	0.61 (0.4–0.8)	0.53 (0.4–0.7)	0.1
Gestational week at blood sample Research Topic (wks)	31 (28–34)	29 (26–33)	32 (29–34)	<0.001
sFlt-1 (pg/mL)	5,684 (2090–11,133)	6,534 (3202–10,915)	5,242 (1647–11,459)	0.3
PlGF (pg/mL)	72.7 (38.8–154.9)	58.1 (30–116.7)	87.1 (45–208.8)	0.008
sFlt-1/PlGF ratio	95.3 (15.8–283.6)	115.6 (34.5–351.7)	75.4 (8.9–259.5)	0.02
Interval between sample Research Topic and delivery (days)	26 (6–47)	17 (4–17)	33 (13–52)	<0.01
Gestational age at delivery (wks)	34 (31–37)	32 (29–34)	37 (37–37)	<0.001
<37 wks*	128 (66.7)	63 (84)	65 (55.6)	<0.001
<34 wks*	90 (46.9)	35 (46.7)	55 (47)	1
<32 wks*	58 (30.2)	32 (42.7)	26 (22.2)	0.004
<30 wks*	35 (18.2)	26 (34.7)	9 (7.7)	<0.001
<28 wks*	19 (9.9)	18 (24)	1 (0.9)	<0.001
Cesarean delivery*	153 (79.7)	58 (77.3)	95 (81.2)	0.5
Neonatal birth weight (g)	1785 (1100–2,190)	1,385 (900–1800)	2,275 (2100–2,405)	<0.001
Apgar <8*	29 (15.1)	21 (28)	8 (6.8)	<0.001
Umbilical blood pH	7.33 (7.3–7.38)	7.33 (7.3–7.37)	7.32 (7.33–7.41)	0.5
NICU*	122 (63.5)	75 (100)	47 (40.2)	<0.001
CPAP*	97 (50.5)	49 (65.3)	48 (41)	0.001
Mechanical ventilation*	31 (16.1)	31 (41.3)	0	<0.001
NEC*	5 (2.6)	5 (6.7)	0	0.008
IVH I/II*	6 (3.10)	3 (4)	3 (2.6)	0.7
IVH III/IV*	1 (0.5)	1 (1.3)	0	0.4
Neonatal death*	10 (5.2)	10 (13.3)	0	<0.001

IQR, interquartile range; ALT, alanine aminotransferase; AST, aspartate *aminotransferase*; LDH, lactate dehydrogenase, PLT, platelet count; UtA, uterine artery pulsatility index; UA PI, umbilical pulsatility index; EADF, absent end-diastolic flow; REDF, reversed end-diastolic flow; MCA PI, middle cerebral artery pulsatility index; DV PIV, ductus venosus pulsatility index; sFlt-1, soluble fms-like tyrosine kinase-1; PlGF, placental growth factor; sFlt-1/PlGF ratio, soluble fms-like tyrosine kinase-1 to placental growth factor ratio; wks, weeks of gestation; NICU, Neonatal Intensive Care Unit hospitalization; CPAP, continuous positive airway pressure; NEC, necrotizing enterocolitis; IVH I/II, intraventricular hemorrhage grade I or II; IVH III/IV, intraventricular hemorrhage grade III or IV. * Number (percentage).

Based on neonatal outcomes, the study group was divided into two subgroups: those with and without adverse outcomes. The characteristics of both groups are presented in Table 1. Adverse outcomes were noted in 75 newborns (39.1%). No significant differences were observed between these groups in terms of maternal age, parity, the occurrence of hypertension, or laboratory findings other than angiogenic biomarkers.

Neonatal outcomes are presented in Table 1. Preterm delivery was reported in 66.7% of cases. Of 90 women who delivered before 34 weeks of gestation, 84 had completed the course of antenatal corticosteroids administered within 7 days before delivery (31 in the adverse outcome groups and 53 in the no adverse outcome group). NICU hospitalization was necessary for 63.5% of infants. Respiratory distress requiring CPAP occurred in 50.5% of cases, and mechanical ventilation was introduced in 16.1% of cases. IVH was diagnosed in 3.6% of cases. Ten newborns died after delivery. Eight of them were born at 25 or 26 weeks’ gestation with a birth weight between 290 and 630 g. One newborn was delivered at 29 weeks with a birth weight of 1,040 g and developed pulmonary hypertension and circulatory failure, while another one was delivered at 30 weeks’ gestation weighing 1,080 g and died of sepsis.

Newborns with AO were delivered earlier and had lower birth weights. Significantly fewer infants in this group were born in good general condition. However, umbilical blood pH did not differ between the groups with and without adverse neonatal outcomes. The incidence of the analyzed pathologies is presented in Table 1.

Significant differences in Doppler evaluation were observed between the groups. In the adverse outcome group, the mean UtA PI and UA PI values were higher. In 10.4% of cases, absent end-diastolic flow in the UA was observed, and in 4.7%, reversed end-diastolic flow in the UA was observed. Conversely, MCA PI and DV PI values were similar in the groups with and without adverse neonatal outcomes.

The serum concentrations of PlGF were lower, leading to a higher sFlt-1/PlGF ratio in the AO group. No significant differences in sFlt-1 levels were observed between the groups. The values are presented in Table 1.

**Figure 2:**
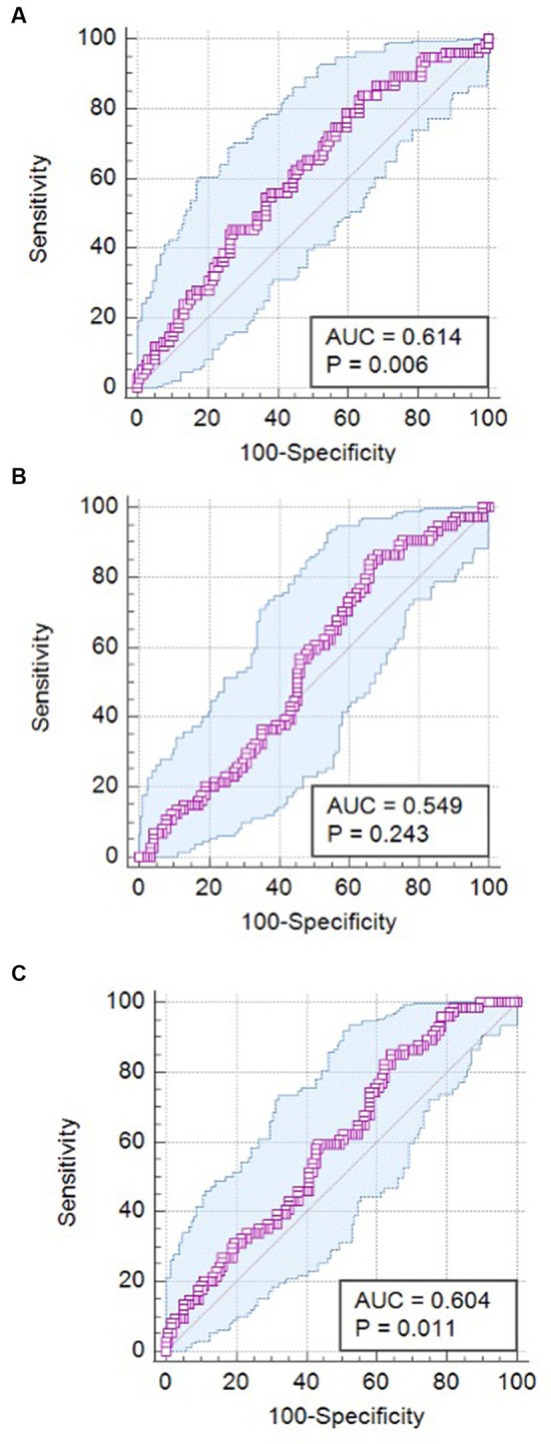
ROC curves for PlGF, sFlt-1, and the sFlt-1/PlGF ratio in the prediction of adverse neonatal outcomes. **(A)** ROC curve for PlGF; **(B)** ROC curve for sFlt-1; **(C)** ROC curve for sFlt-1/PlGF.

The cutoff points for sFlt-1, PlGF, and the sFlt-1/PlGF ratio in the prediction of adverse neonatal outcomes were determined based on ROC curves. The ROC curves are presented in Figure 2, and the sensitivity, specificity, and negative and positive likelihood ratios are presented in Table 2. The sFlt-1/PlGF ratio exhibited high sensitivity but low specificity in predicting adverse neonatal outcomes, with an area under the curve of 0.604 (*p* = 0.011).

**Table 2:** Sensitivity, specificity, and positive and negative likelihood ratios for PlGF, sFlt-1, and the sFlt-1/PlGF ratio in the prediction of adverse neonatal outcomes.

Cutoff values	Sensitivity (95% CI)	Specificity (95% CI)	Positive likelihood ratio (95% CI)	Negative likelihood ratio (95% CI)	PPV (95% CI)	NPV (95% CI)
PlGF = 137	84 (73.7–91.4)	35.9 (27.2–45.3)	1.31 (1.11–1.55)	0.45 (0.25–0.79)	45.7 (41.5–49.8)	77.8 (66.4–86.1)
sFlt-1 = 2,236	85.1 (75–92.3)	33.3 (24.9–42.6)	1.28 (1.09–1.5)	0.45 (0.24–0.81)	44.7 (40.8–48.7)	78 (66.0–86.6)
sFlt-1/PlGF ratio = 19.1	85.1 (75–92.3)	35.9 (27.2–45.3)	1.33 (1.13–1.57)	0.41 (0.23–0.75)	45.7 (41.6–49.8)	79.2 (67.8–87.4)

95% CI, 95% confidence interval; PlGF, placental growth factor; sFlt-1, soluble fms-like tyrosine kinase-1; sFlt-1/PlGF ratio, soluble fms-like tyrosine kinase-1 to placental growth factor ratio.

Spearman’s correlation analysis between the analyzed variables was performed. Both PlGF and sFlt-1 had a moderate correlation with adverse neonatal outcomes (PlGF: R − 0.5, *p* < 0.001; sFlt-1: 0.5, *p* < 0.001). The sFlt-1/PlGF ratio showed a correlation of 0.6 (*p* < 0.001) with AO. Spearman’s rank correlation coefficients for specific neonatal outcomes are presented in Table 3.

**Table 3:** Spearman’s rank correlation coefficients for the analyzed variables.

	AO	NICU	Mechanical ventilation	NEC	IVH III/IV	Neonatal death	Gestational age at delivery	Birth weight
PlGF	−0.5 *p* < 0.001	−0.5 *p* < 0.001	−0.3 p < 0.001	−0.2 *p* = 0.01	−0.1 *p* = 0.2	−0.2 *p* = 0.01	0.6 *p* < 0.001	0.6 *p* < 0.001
sFlt-1	0.5 *p* < 0.001	0.5 *p* < 0.001	0.2 *p* = 0.03	0.1 *p* = 0.1	0.1 *p* = 0.09	0.002 *p* = 0.9	−0.4 *p* < 0.001	−0.4 *p* < 0.001
sFlt-1/PlGF ratio	0.6 *p* < 0.001	0.5 *p* < 0.001	0.3 *p* < 0.001	0.2 *p* = 0.003	0.1 *p* = 0.09	0.1 *p* = 0.09	−0.6 *p* < 0.001	−0.6 *p* < 0.001

PlGF, placental growth factor; sFlt-1, soluble fms-like tyrosine kinase-1; sFlt-1/PlGF ratio, soluble fms-like tyrosine kinase-1 to placental growth factor ratio; NICU, Neonatal Intensive Care Unit hospitalization; NEC, necrotizing enterocolitis; IVH III/IV, intraventricular hemorrhage grade III or IV.

Neonatal birth weight, gestational week at delivery, UtA PI, UA PI, DV PI, PlGF, sFlt-1, and sFlt-1/PlGF ratio were taken into account in the logistic regression analysis for adverse neonatal outcomes. Among the analyzed variables, only the UtA PI and the sFlt-1/PlGF ratio were independent risk factors for adverse outcomes. The results are presented in Table 4.

**Table 4:** Logistic regression analysis for adverse neonatal outcomes.

Variable	Adjusted OR	95% CI	*p*
Birth weight	0.99	0.99 to 1.00	0.3
Gestational age at delivery	0.96	0.71 to 1.30	0.8
sFlt-1/PlGF ratio	0.99	0.99 to 1.00	0.04
UtA PI	2.63	1.15 to 6.03	0.02
UA PI	0.66	0.29 to 1.47	0.3

OR, odds ratio; sFlt-1/PlGF ratio, soluble fms-like tyrosine kinase-1 to placental growth factor ratio; UtA PI, uterine artery pulsatility index; UA PI, umbilical artery pulsatility index.

## Discussion

In this prospective study of 192 SGA newborns, significant differences were found in the PlGF and sFlt-1/PlGF ratio values between infants with and without adverse outcomes, while no differences were observed in the sFlt-1 concentrations. In logistic regression analysis, only the UtA PI and the sFlt-1/PlGF ratio were identified as independent risk factors for AOs. An sFlt-1/PlGF ratio of 19.1 exhibited high sensitivity (85.1%) but low specificity (35.9%) in predicting adverse outcomes, and had the strongest correlation with them. This ratio allowed for the assessment of the risk of AOs to be low, with almost 80% certainty..

Angiogenic biomarkers in pregnancies complicated by fetal growth abnormalities have been investigated by other researchers. Mendoza et al. conducted a prospective observational study of singleton pregnancies with EFW below the 10th centile between 20 + 0 and 31 + 6 weeks of gestation. The authors aimed to investigate the capacity of a predictive model to assess individual risks for prenatal counseling at the time of diagnosis. They included 49 SGA fetuses and 124 with FGR. The median value of sFlt-1/PlGF in a cohort of women diagnosed with SGA fetuses was 9.7 pg./mL (IQR 3.2–125.2) (11). In our study, the median value of sFlt-1/PlGF was 95.3 (IQR 15.8–283.6), which is approximately 10 times higher than in the aforementioned study. The values presented by other authors also vary. In the study by Dymara-Konopka et al., the median value of sFlt-1/PlGF was 219 (IQR 81–846) in an isolated FGR group, while Garcia-Manau et al. found it to be 4.14 (IQR 2.12–7.42) (12, 13). In the study by Shim et al., the mean value of sFlt-1/PlGF was 28.62 ± 38.4 (14). It is possible that the results vary due to the heterogeneity of the studied populations, as fetal growth abnormalities may be caused by various etiologic factors (15, 16).

The utility of sFlt-1/PlGF in the prediction of AO in SGA newborns has been investigated in several studies. Mendoza et al. found AO in 55.9% of all SGA infants. Adverse outcomes included stillbirth (1.7%), neonatal death (2.9%), respiratory distress syndrome (23.7%), sepsis (7.5%), NEC (2.9%), IVH grade III or IV (1.2%), Apgar score < 7 points (14.5%), and umbilical artery blood pH ≦7.0 (2.3%) (11). Those results remain in accordance with ours. According to Mendoza et al., the sFlt-1/PlGF ratio was the single biomarker with the largest AUC for the prediction of adverse perinatal outcomes (AUC 0.833, 95% CI 0.77–0.9), with an adjusted odds ratio of 1.005 (95% CI 1.002–1.01). The sFlt-1/PlGF ratio alone had similar characteristics in the prediction of AOs as a multivariable model, including UA and UtA Doppler assessment, EFW centile, and gestational age at delivery. As the simplest tool should be recommended in clinical practice, the authors suggested using the sFlt-1/PlGF ratio alone for the personalized prediction of adverse outcomes (11). In our study, the sFlt-1/PlGF ratio and the UtA PI were the only two independent risk factors for AOs. Therefore, we agree with Mendoza et al. that the sFlt-1/PlGF ratio alone could be an efficient clinical tool for predicting adverse neonatal outcomes in SGA newborns. It is also possible that the value of the sFlt-1/PlGF ratio correlates with the grade of AO. For example, Garcia-Manau et al. found a significant correlation between the sFlt-1/PlGF ratio and length of NICU hospitalization (R 0.311, 95% CI 0.095–0.499, *p* = 0.006) in SGA newborns (13). As this is a very interesting issue with possible clinical implications, more research is needed on this issue.

Shim et al. investigated the utility of angiogenic biomarkers in the prediction of adverse neonatal outcomes in SGA newborns in normotensive women. Adverse outcomes requiring NICU admission were associated with subcauses, such as jaundice, meconium aspiration syndrome, transient tachypnea of the newborn, respiratory distress syndrome, NEC, sepsis, and need for mechanical ventilation. The authors analyzed the sFlt-1/PlGF values at 24 to 28 + 6 weeks and at 29 to 36 + 6 weeks of gestation. The angiogenic biomarkers assessed at 24 to 28 + 6 weeks had no prognostic value, while the cutoff point for the sFlt-1/PlGF ratio of 28.15 measured between 29 and 36 + 6 weeks of gestation had a sensitivity of 76.9% and a specificity of 88% (AUC area: 0.907, 95% CI 0.829–0.985) in the prediction of AOs (14). In our study, with the cutoff point at 19.1, the sensitivity was higher while the specificity was lower, which may be related to the exclusion of hypertensive women in the study by Shim et al. They calculated the adjusted odds ratio for the sFlt-1/PlGF ratio to be 1.017 (95% CI 1.004–1.030), which was similar to our results (14).

Bonacina et al. investigated a cohort of 175 singleton pregnancies with EFW below the 10th centile between 20 and 31 + 6 weeks. They defined AOs as stillbirth, cesarean section for non-reassuring CTG, or any adverse neonatal outcome (neonatal death, respiratory distress syndrome, bronchopulmonary dysplasia, neonatal sepsis, retinopathy of prematurity stage III–IV, NEC, IVH grade III–IV, periventricular leukomalacia, 5 min Apgar score < 5, or UA cord pH <7). For the prediction of adverse perinatal outcomes in SGA, the greatest AUC (0.763, 95% CI 0.699–0.826) was achieved with the sFlt-1/PlGF cutoff of 24.9. The cutoff of 24.9 showed a negative predictive value of 75.9% to rule out and a positive predictive value of 79.1%. The authors observed that this cutoff was not significantly superior to the one of 38 for ruling in and ruling out AO. Therefore, the authors recommended the one with the greater negative predictive value. Consequently, the cutoff of 38 would be the most suitable for AO. In our study, the cutoff of 19.1 had a sensitivity of 85.1%, a specificity of 35.9%, a positive predictive value of 45.7%, and a negative predictive value of 79.2%. Conversely, the cutoff of 38 in our study had a sensitivity of 74.3%, a specificity of 41.9%, a positive predictive value of 44.7% (95% CI 39.7–49.8), and a negative predictive value of 72.1% (95% CI 62.4–80.1). Therefore, based on our data, the sFlt-1/PlGF ratio of 19.1 had the highest negative predictive value and could be used to counsel parents on the risk of AOs in cases of SGA diagnosis.

Angiogenic biomarkers were previously assessed to predict outcomes related to delivery in SGA newborns. Sharp et al. observed that the combination of clinical biometric data and the sFlt-1/PlGF ratio could predict pregnancy outcomes for live birth, gestational age at delivery, birth weight, and overall survival in early-onset FGR (17). Miranda et al. reported that the combination of fetal weight, UA PI, cerebro-placental ratio, estriol, and PlGF predicted 62% of adverse outcomes in SGA cases with a false positive rate of 10%. In their study, AO was defined as the occurrence of stillbirth, umbilical artery cord blood pH <7.15, 5 min Apgar score < 7, or emergency operative delivery for fetal distress (18). Dymara-Konopka et al. conducted a prospective cross-sectional case–control study to assess a potential relationship between the concentration of sFlt-1, PlGF, sFlt-1/PlGF ratio, maternal or fetal Doppler flow measurements, and perinatal outcomes in pregnancies complicated by FGR with and without preeclampsia (PE). Although the study group was small (14 cases of isolated FGR), the authors found a positive correlation between PlGF and the Apgar score at 1 and 5 min in the FGR group (12). As these biomarkers are now widely used, their usefulness in the prediction of adverse neonatal outcomes would constitute an added value in clinical practice.

The sFlt-1/PlGF ratio seems to be efficient in the prediction of AOs in SGA newborns. However, the same effect may not concern the normal population. Valiño et al. investigated a cohort of 3,953 singleton pregnancies at 35–37 weeks gestation and found no differences in PlGF, sFlt-1, or the sFlt-1/PlGF ratio values between women delivering newborns with adverse or normal outcomes (defined as low cord blood pH, NICU admission, or low 5 min Apgar score) (19).

The strengths of our study include its prospective and multicenter design and a relatively large cohort of SGA newborns managed in the same way, with complete medical data and available angiogenic biomarker results. As we performed PlGF and sFlt-1 blood concentration measurements at the time of SGA diagnosis, our results may be useful for personalized counseling of parents diagnosed with SGA fetuses during pregnancy. Therefore, the study provides valuable information for individualized assessment from the time of SGA diagnosis. Moreover, as we included pregnancies above 24 weeks of gestation, we adjusted the AO diagnosis to gestational age to make it a more clinically useful definition. However, there are some limitations to the study. Computerized CTG was not used in the whole cohort, so it was not included in the analysis. Since genetic or major anatomical abnormalities in the fetus were excluded, our conclusions cannot be used in such circumstances. The cohort of all SGA fetuses is heterogeneous, as it includes fetuses that are constitutionally small and growth-restricted. However, from a clinical point of view, it is sometimes challenging to differentiate between FGR and SGA, as the definitions are based on ultrasound measurements, which are subject to human error. Therefore, we believe that it is clinically relevant to investigate various biomarkers to determine their usefulness in predicting adverse outcomes and their potential to assist in counseling patients. Generalization and investigation of all cases of SGA may, in fact, facilitate the selection of clinical management.

Proper prenatal counseling on AO in SGA newborns is essential. The sFlt-1/PlGF ratio seems to be an efficient predictive tool in adverse outcome risk assessment. More studies on large cohorts of SGA-complicated pregnancies regarding preeclampsia, chromosomal and anatomical abnormalities, and intrauterine infections are needed to develop an optimal and detailed formula for the risk assessment of AO in SGA newborns.

## Data availability statement

The raw data supporting the conclusions of this article will be made available by the authors, without undue reservation.

## Ethics statement

The studies involving humans were approved by Ethics Committee at the Center of Postgraduate Medical Education. The studies were conducted in accordance with the local legislation and institutional requirements. The participants provided their written informed consent to participate in this study.

## Author contributions

KK-K: Conceptualization, Formal analysis, Investigation, Methodology, Writing – original draft. KC: Data curation, Writing – original draft. NS-S: Data curation, Writing – original draft. RB-B-S: Visualization, Writing – original draft. AC: Data curation, Writing – original draft. KŻ: Data curation, Writing – original draft. ND: Writing – review & editing. JM: Data curation, Writing – original draft. JG: Data curation, Writing – original draft. KB: Data curation, Writing – original draft. WW: Data curation, Writing – original draft. AS: Writing – review & editing.
